# Microaneurysms detection in color fundus images using machine learning based on directional local contrast

**DOI:** 10.1186/s12938-020-00766-3

**Published:** 2020-04-15

**Authors:** Shengchun Long, Jiali Chen, Ante Hu, Haipeng Liu, Zhiqing Chen, Dingchang Zheng

**Affiliations:** 1 College of Computer Science and Technology, Zhejiang University of Technology, Hangzhou, 310023 China; 2 Research Center of Intelligent Healthcare, Faculty of Health and Life Science, Coventry University, Coventry, CV1 5RW UK; 3 Eye Center, The second Affiliated Hospital of Zhejiang University School of Medicine, Hangzhou, 310009 China

**Keywords:** Color fundus image, Microaneurysms’ detection, Patch, Feature extraction, Directional local contrast, Machine learning

## Abstract

**Background:**

As one of the major complications of diabetes, diabetic retinopathy (DR) is a leading cause of visual impairment and blindness due to delayed diagnosis and intervention. Microaneurysms appear as the earliest symptom of DR. Accurate and reliable detection of microaneurysms in color fundus images has great importance for DR screening.

**Methods:**

A microaneurysms' detection method using machine learning based on directional local contrast (DLC) is proposed for the early diagnosis of DR. First, blood vessels were enhanced and segmented using improved enhancement function based on analyzing eigenvalues of Hessian matrix. Next, with blood vessels excluded, microaneurysm candidate regions were obtained using shape characteristics and connected components analysis. After image segmented to patches, the features of each microaneurysm candidate patch were extracted, and each candidate patch was classified into microaneurysm or non-microaneurysm. The main contributions of our study are (1) making use of directional local contrast in microaneurysms' detection for the first time, which does make sense for better microaneurysms' classification. (2) Applying three different machine learning techniques for classification and comparing their performance for microaneurysms' detection. The proposed algorithm was trained and tested on e-ophtha MA database, and further tested on another independent DIARETDB1 database. Results of microaneurysms' detection on the two databases were evaluated on lesion level and compared with existing algorithms.

**Results:**

The proposed method has achieved better performance compared with existing algorithms on accuracy and computation time. On e-ophtha MA and DIARETDB1 databases, the area under curve (AUC) of receiver operating characteristic (ROC) curve was 0.87 and 0.86, respectively. The free-response ROC (FROC) score on the two databases was 0.374 and 0.210, respectively. The computation time per image with resolution of 2544×1969, 1400×960 and 1500×1152 is 29 s, 3 s and 2.6 s, respectively.

**Conclusions:**

The proposed method using machine learning based on directional local contrast of image patches can effectively detect microaneurysms in color fundus images and provide an effective scientific basis for early clinical DR diagnosis.

## Background

Diabetes mellitus has reached epidemic levels worldwide. In 2019, approximately 463 million adults (20–79 years) were living with diabetes; by 2045 this will rise to 700 million [1]. Diabetic eye disease (DED) is one of the serious complications caused by diabetes, which can cause serious effects on eyes [2]. Most importantly, diabetic retinopathy (DR) is the leading cause of visual impairment and blindness among working age adults. Among people with diabetes, the incidence of DR is approximately one-third [3]. Clinical evidence shows that early diagnose and clinical intervention of DR can effectively reduce the risk of DR-related vision loss [3].

At present, diabetic eye disease (especially DR) is clinically diagnosed using fundus imaging techniques [4], mainly including fundus photography, fundus fluorescein angiography (FFA), and optical coherence tomography (OCT) [5]. All the imaging techniques were widely used in automatic detection of DR. As described in reviews of fundus imaging techniques [4, 6], compared with others, fundus photography can be used to document retinal disease over time, and may be increasingly helpful in screening of DR.

**Fig. 1:**
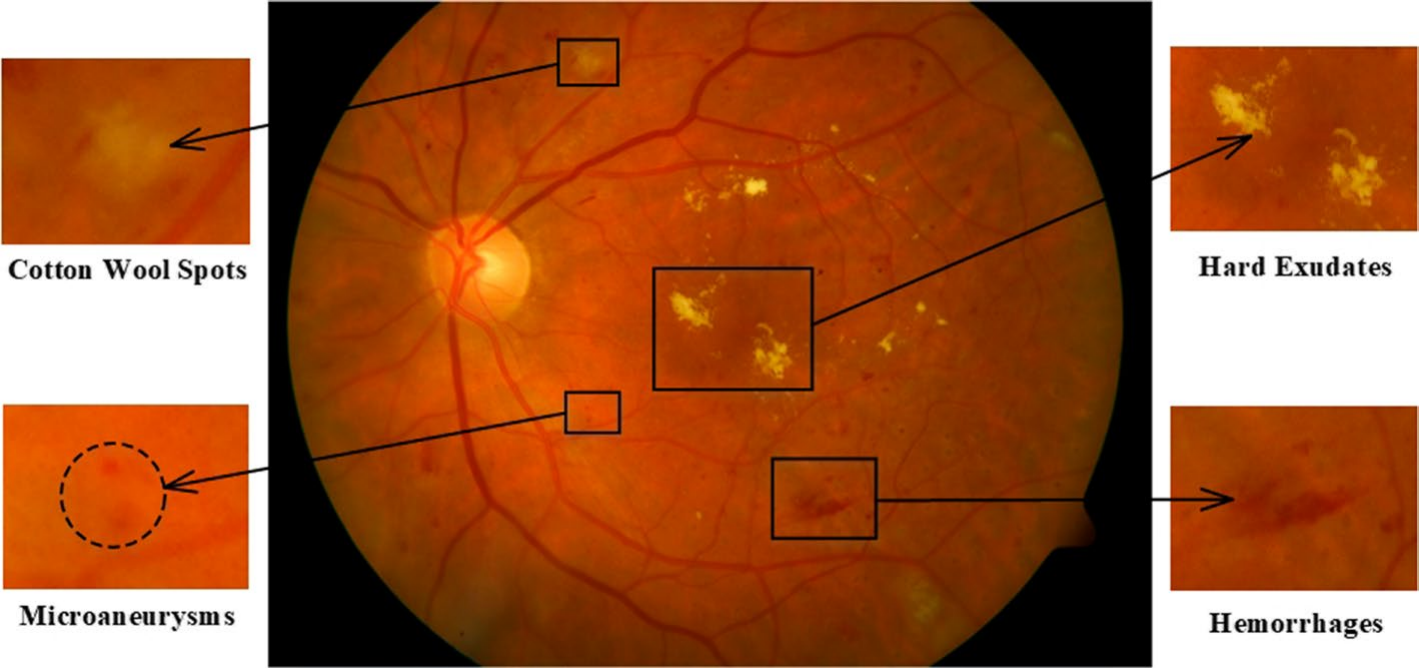
Color fundus image of DR

Therefore, color fundus images obtained from fundus photography were used in this study, which of DR is shown in Fig. 1. Retinal abnormalities caused by DR can be observed from Fig. 1, including microaneurysms (MAs), hemorrhages (HMs), hard exudates (HEs) and cotton wool spots (CWSs). MAs often appear in the fundus posterior pole and as red or dark red isolated small dots with clear borders in color fundus images. The diameter of MA is usually between 15 and $60\upmu m$ [7], or larger, but seldom exceeds $125\upmu m$ [8]. Compared with other DR-related lesions such as HE and CWS, MA is difficult to detect due to its tiny structure.

MA is the earliest symptom of DR, which is an important symptom in DR progression. In clinical, MA count is associated with DR severity [9], and MA formation rate concerns the development of clinically significant macular edema (CSME) in patients with mild-to-moderate non-proliferative DR (NPDR); patients with more MA formation rate having higher risk to develop CSME [10]. It is considered that MA turnover correlates with Early Treatment Diabetic Retinopathy Study (ETDRS) grading worsening and development of central-involved macular edema (CIME) [11]. Therefore, automatic and reliable detection of MA in color fundus images using computer-aided techniques is significant for the early screening and diagnosis of DR, which also contributes to the identification of those eyes at risk of developing CSME [10].

Computer-aided automatic analysis of color fundus images is highly efficient and has been widely applied in the diagnosis of DR [12–15], which could reduce the workload of ophthalmologists and improve the efficiency of DR screening. With the development of computer-aided diagnose (CAD) of DR, due to the importance of MA for DR diagnose, there are more and more studies on automatic detection of MA recently. Saha et al. [16] detected red lesions and bright lesions using Naive Bayesian (NB) classifier and support vector machine (SVM), but they only used one database including 100 images for both training and testing. Seoud et al. [17] proposed a method based on random forest (RF) for the detection of both MA and HM using dynamic shape features, which did not need any prior segmentation of lesions. But the lesions linked to blood vessels were missed leading to false-negatives (FN) [15]. Srivastava et al. [18] proposed filters on different grid sizes and combined with multiple-kernel learning (MKL) and SVM to detect MA and HM dealing with the false-positives (FP) due to small blood vessel (BV) segments. Using MKL was found to improve the performance as compared to using a single grid size, but with a disadvantage of high computations for higher grid size. Zhou et al. [19] proposed an unsupervised classification method based on sparse principal component analysis (PCA) for MA detection, which can avoid the class imbalance problem. But there are some FPs during feature extraction. Ren et al. [20] detected MA by adaptive over-sampling boosting (ADOBoosting), while Dai et al. [21] and Dashtbozorg et al. [22] applied the random under-sampling boosting (RUSBoosting) classifier to detect MA, and all of them performed well with respect to class-imbalance problem. Wu et al. [23] used peak detection and region growing to get MA candidates, then detected MA based on K-nearest neighbor (KNN), with a relatively low FROC score of 0.273 on e-ophtha MA database. Wang et al. [24] proposed a method using singular spectrum analysis and KNN classifier for MA detection, which obtained some FNs due to missing of subtle or low contrast or blurry-outlined MA. They also missed few MA during candidate extraction. Adal et al. [25] presented a multi-stage approach for automated detection of longitudinal retinal changes due to MA and dot HM (small red lesions). Derwin et al. [26] detected MA by applying local binary pattern (LBP) for texture features’ extraction and followed by SVM for classification. Javidi et al. [27] and Wei et al. [28] detected MA by discriminative dictionary learning (DDL) and multi-feature fusion dictionary learning (MFFDL), respectively. The former depended heavily on original grayscale feature dictionary, and since there is a large variability in color, luminosity, and contrast both within and between retinal images, using single grayscale feature will affect the performance.

As deep learning is an emerging computer vision application in medical image processing and proving to be of great help to mankind in machine learning [15], several MA detection methods based on convolutional neural networks (CNN) [8, 29–32] were proposed. The main limitation of CNN is the requirement of larger training time [15].

Moreover, some studies mentioned above for MA detection only used one database for training and testing [19, 25–28, 32]. Large-scale data on heterogeneous patient cohorts are needed for full validation. Considering the limitations aforementioned, mainly improvement of MA detection results, lower computation time and enough data for validation are mainly required.

We aim to propose a new method using machine learning algorithms based on directional local contrast (DLC) feature for MA detection, and validate it on two different databases. The first step is preprocessing to improve the image quality, including segmentation and removal of blood vessel (BV). An improved enhancement method based on Hessian matrix eigenvalue analysis was used for BV segmentation. With main structure of BV eliminated, MA candidates were extracted based on shape characteristics and connected components’ analysis. Next, features were extracted from each candidate to differentiate the MA and non-MA. Last, machine learning methods were applied for classification based on the extracted features and comparing their performances on MA detection.

**Fig. 2:**
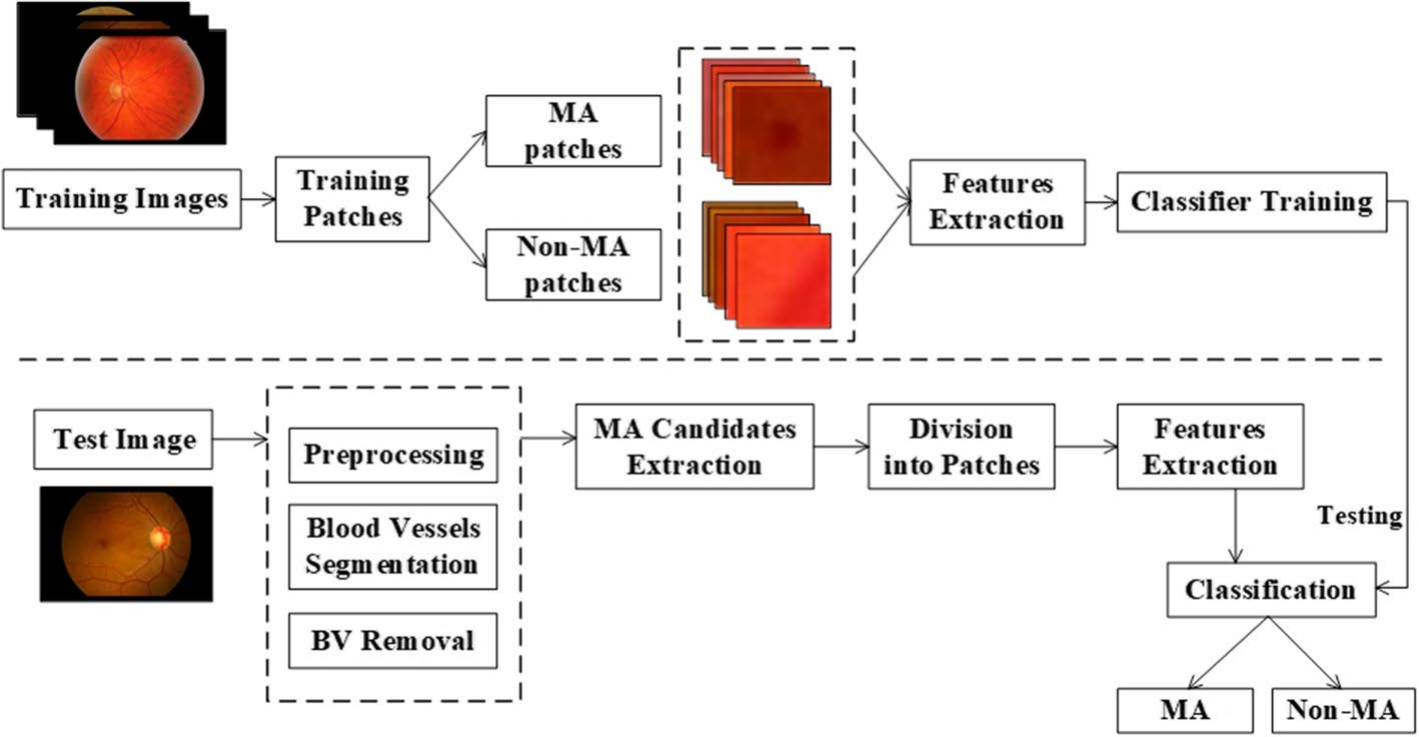
Illustration of the proposed MA detection method

The main contributions of our study are (1) using DLC feature which has not been used in existing methods for MA detection. DLC indicates the local contrast characteristics in a neighborhood which was applied as feature to distinguish MA, and it was proven that the method performs better using DLC than not. (2) Applying three different machine learning methods for MA classification and comparing their performance, especially, Naive Bayesian classification which is simple with low computational complexity and efficient on accuracy and computation time. The overview of the proposed method is depicted in Fig. 2.

The remaining paper is organized as follows:“Results” section presents the results of our proposed method. In “Discussion” and “Conclusions” sections, discussion and conclusions are presented. The proposed method is described in “Method” section including experiment and evaluation measures.

## Results

Results of the proposed method were evaluated on lesion level, including ROC curves of the three classifiers, computation time, and comparison with existing methods on FROC curve and time.

### ROC curves

**Fig. 3:**
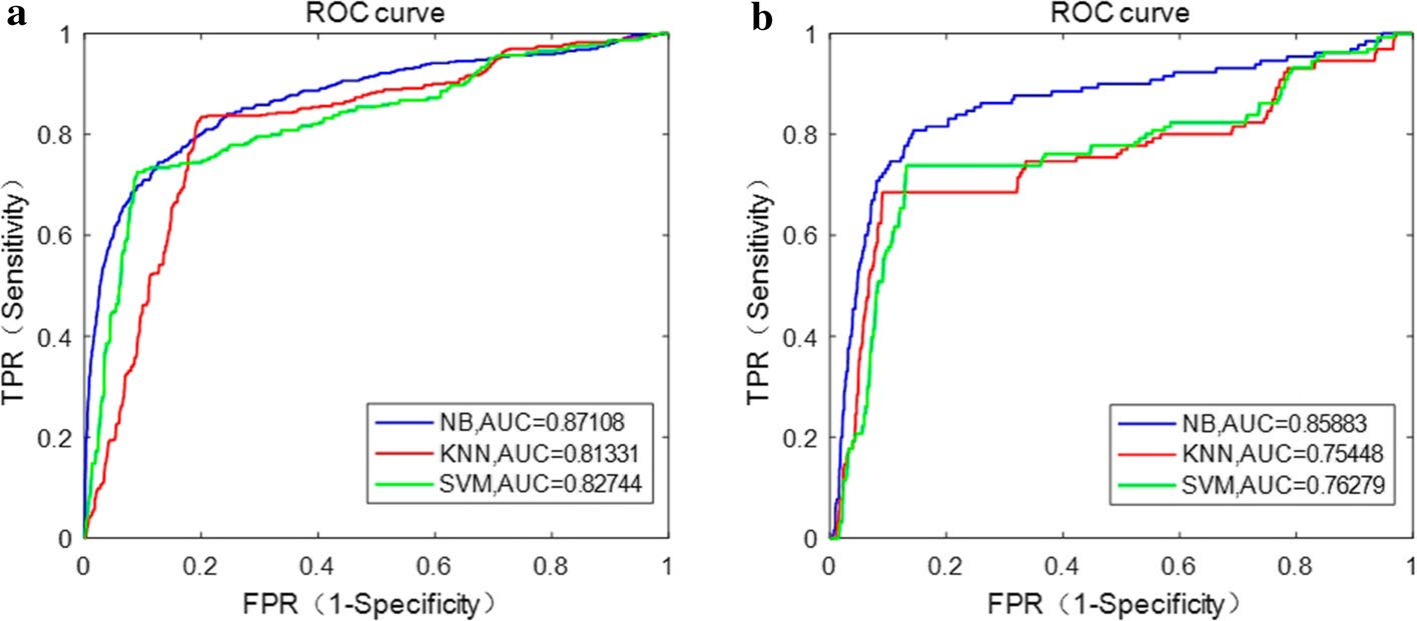
ROC curves of three classifiers on the two databases. **a** e-ophtha MA database. **b** DIARETDB1 database

The ROC curve and corresponding AUC of MA detection results on e-ophtha MA and DIARETDB1 databases achieved by three classifiers (Naive Bayesian, KNN and SVM) are shown in Fig. 3.

**Fig. 4:**
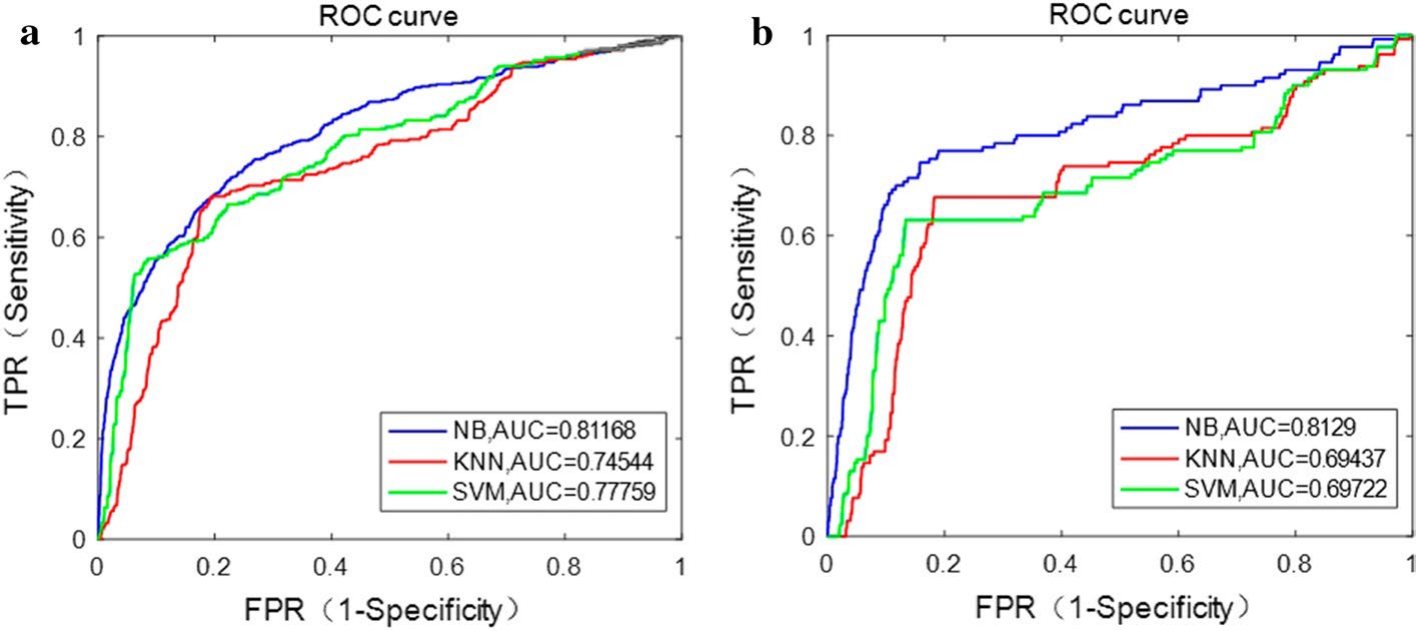
ROC curves of three classifiers on the two databases without using DLC feature. **a** e-ophtha MA database. **b** DIARETDB1 database

In this work, the directional local contrast of the center point *p* of each MA candidate patch was calculated, including totally 12 values of different directions of DLC. Without using the DLC feature, the results of three classifiers are shown in Fig. 4. As can be seen from Figs. 3 and 4, the proposed method performs better using DLC feature compared with not.

It can be seen that Naive Bayesian classifier performs better than SVM and KNN, with the AUC of 0.871 and 0.859 on e-ophtha MA and DIARETDB1 databases, respectively. Therefore, Naive Bayesian was selected for evaluation of MA detection performance in this study.

### Computation time

To run the proposed method for MA detection, MATLAB 2016a (The MathWorks, Inc., Natick, Massachusetts, USA) was used in the environment of 64-bit Windows 10 operating system with 2.9 GHz Intel Core i5 CPU and 16GB memory.

The computation time mainly depends on image resolution and the number of MAs labeled per image. The average computation time per image in the test set of e-ophtha MA database and DIARETDB1 database is shown is Table 1. This means the larger the resolution is, the more the computation time is. The more the MAs labeled per image, the greater the number of extracted MA candidates, resulting in more computation time for making candidate patches, feature extraction and classification. The average time per image in each processing step is shown in Table 2 and compared with that of Dashtbozorg et al. [22]

**Table 1:** Average computation time per image

Databases	Resolutions	Number of MAs labeled per image	Computation time per image
e-ophtha MA	$2544\times 1696$	7.38 (273 in 37 images)	$\sim 29$ s
	$1400\times 960$	8.16 (301 in 37 images)	$\sim 3$ s
DIARETDB1	$1500\times 1152$	2.04 (182 in 89 images)	$\sim 2.6$ s

**Table 2:** Average time per image in each processing step

Steps	Average time (ms) of different resolutions			Average time of Dashtbozorg [22]
	${2544}\times {1696}$	${1440}\times {960}$	${1500}\times {1152}$	
Preprocessing	$\sim 4409$	$\sim 1229$	$\sim 2528$	$\sim 11.0$ s
MA Candidate extraction	$\sim 14997$	$\sim 701$	$\sim 75$	$\sim 52.5$ s
Making candidate patch	$\sim 222$	$\sim 33$	$\sim 14$	−
Feature extraction	$\sim 4591$	$\sim 561$	$\sim 17$	$\sim 98.4$ s
Classification	$\sim 4599$	$\sim 568$	$\sim 5$	$\sim 18.1$ s

### Comparison with different methods

**Fig. 5:**
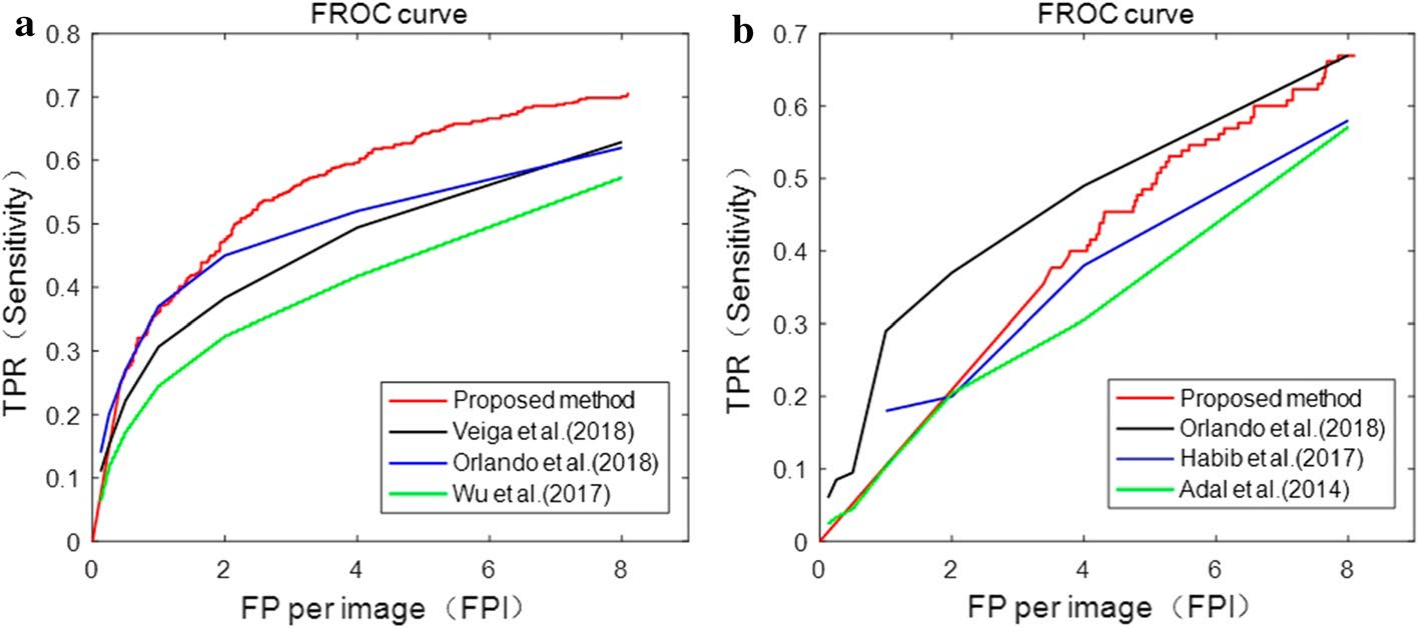
FROC curves of different methods for MA detection on the two databases. **a** e-ophtha MA database. **b** DIARETDB1 database

Figure 5 shows FROC curves of the proposed MA detection method on e-ophtha MA and DIARETDB1 databases compared with existing algorithms.

Tables 3 and 4 show comparison of Sensitivity between the proposed MA detection method and existing algorithms at different FPI values on e-ophtha MA and DIARETDB1 databases, respectively.

**Table 3:** Comparison of sensitivity at different FPI values for different MA detection methods on e-ophtha MA database

Methods	Highlights	Sensitivity under different FPI values							Scores
		$\frac{{1}}{{8}}$	$\frac{{1}}{{4}}$	$\frac{{1}}{{2}}$	1	2	4	8	
Eftekhari [8]	2-step CNN	0.091	0.258	0.401	0.534	0.579	0.667	0.771	0.471
Veiga [39]	Laws texture masks, SVM	0.110	0.152	0.222	0.307	0.383	0.494	0.629	0.328
Orlando [42]	CNN-based features, RF	0.14	0.20	0.23	0.37	0.45	0.52	0.62	0.361
Wu [23]	Profile features, KNN	0.063	0.117	0.172	0.245	0.323	0.417	0.573	0.273
Proposed method	DLC feature, NB	0.075	0.154	0.267	0.358	0.472	0.594	0.699	0.374

**Table 4:** Comparison of sensitivity at different FPI values for different MA detection methods on DIARETDB1 database

Methods	Highlights	Sensitivity under different FPI values							Scores
		$\frac{{1}}{{8}}$	$\frac{{1}}{{4}}$	$\frac{{1}}{{2}}$	1	2	4	8	
Chudzik [31]	Fully convolutional neural networks (FCN)	0.187	0.246	0.288	0.365	0.449	0.570	0.641	0.392
Orlando [42]	CNN-based features, RF	0.06	0.09	0.10	0.29	0.37	0.49	0.67	0.294
Habib [38]	Tree ensemble	−	−	−	0.18	0.20	0.38	0.58	0.2109
Adal [25]	Semi-supervised learning	0.024	0.033	0.045	0.103	0.204	0.305	0.571	0.184
Proposed method	DLC feature, NB	0.013	0.026	0.052	0.104	0.209	0.400	0.669	0.210

Table 5 shows comparison of average computation time for different methods.

**Table 5:** Comparison of computation time for different MA detection methods

Methods	Highlights	Average time	Databases used
Derwin [26]	Texture descriptors, SVM	29 s	One database, in resolutions of $768\times 576$, $1058\times 1061$ and $1389\times 1383$
Chudzik [31]	FCN	220 s	e-ophtha MA and DIARETDB1, with FROC score of 0.562 and 0.369
Dashtbozorg [22]	Local convergence index features, RUSBoosting	3 min	e-ophtha MA and DIARETDB1, with FROC score of 0.546 and 0.547
Wang [24]	Singular spectrum analysis, KNN	1 min	DIARETDB1 database, with Sensitivity of 0.517 at 1 FPI
Habib [38]	Tree ensemble	65 s	DIARETDB1 database, with FROC score of 0.2109
Seoud [17]	Dynamic shape features, RF	98 s for range of 2000–3000 pixels	DIARETDB1 database, with Sensitivity of 0.6 at 6 FPI
Proposed method	DLC feature, NB	29 s for $2544\times 1696$, 3 s for $1400\times 960$, 2.6 s for $1500\times 1152$	e-ophtha MA and DIARETDB1, with FROC score of 0.374 and 0.210

It can be seen that the proposed method has better performance on MA detection compared to existing algorithms. Especially, as to those using deep learning techniques, the algorithms proposed by Eftekhari et al. [8] and Chudzik et al. [31] performed very well on FROC score, using two-step CNN and FCN, respectively. It is known that deep learning with CNN for classification has a better performance on accuracy but more computation time for training, requirement of a large number of training samples and higher requirements for experiment equipment (best with GPU).

### Typical examples’ analysis

**Fig. 6:**
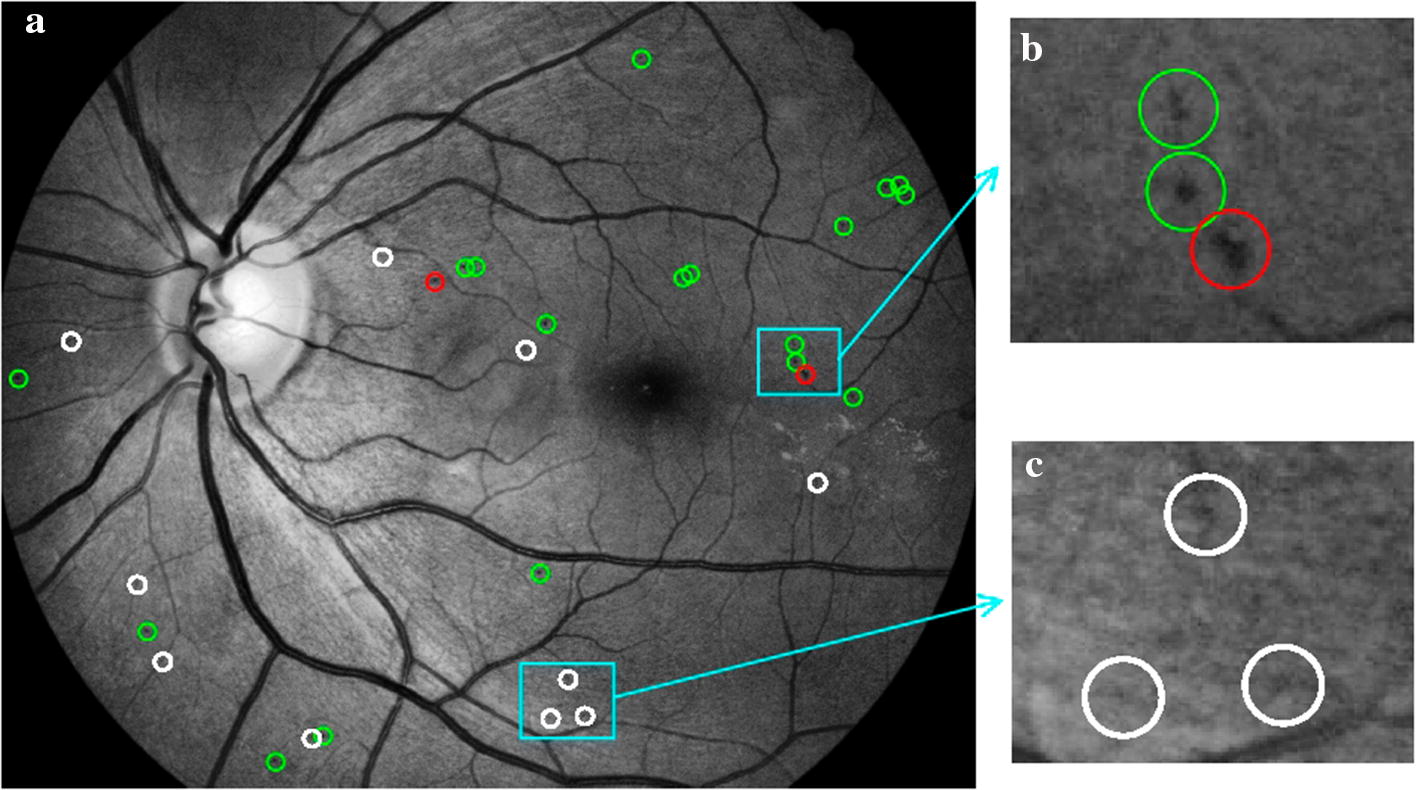
Lesion level evaluation for MA detection results on e-ophtha MA database. **a** Results of MA detection, where green circles indicate TPs, white circles indicate FPs, and red circles indicate FNs; **b** examples of TP and FN; **c** examples of FP

**Fig. 7:**
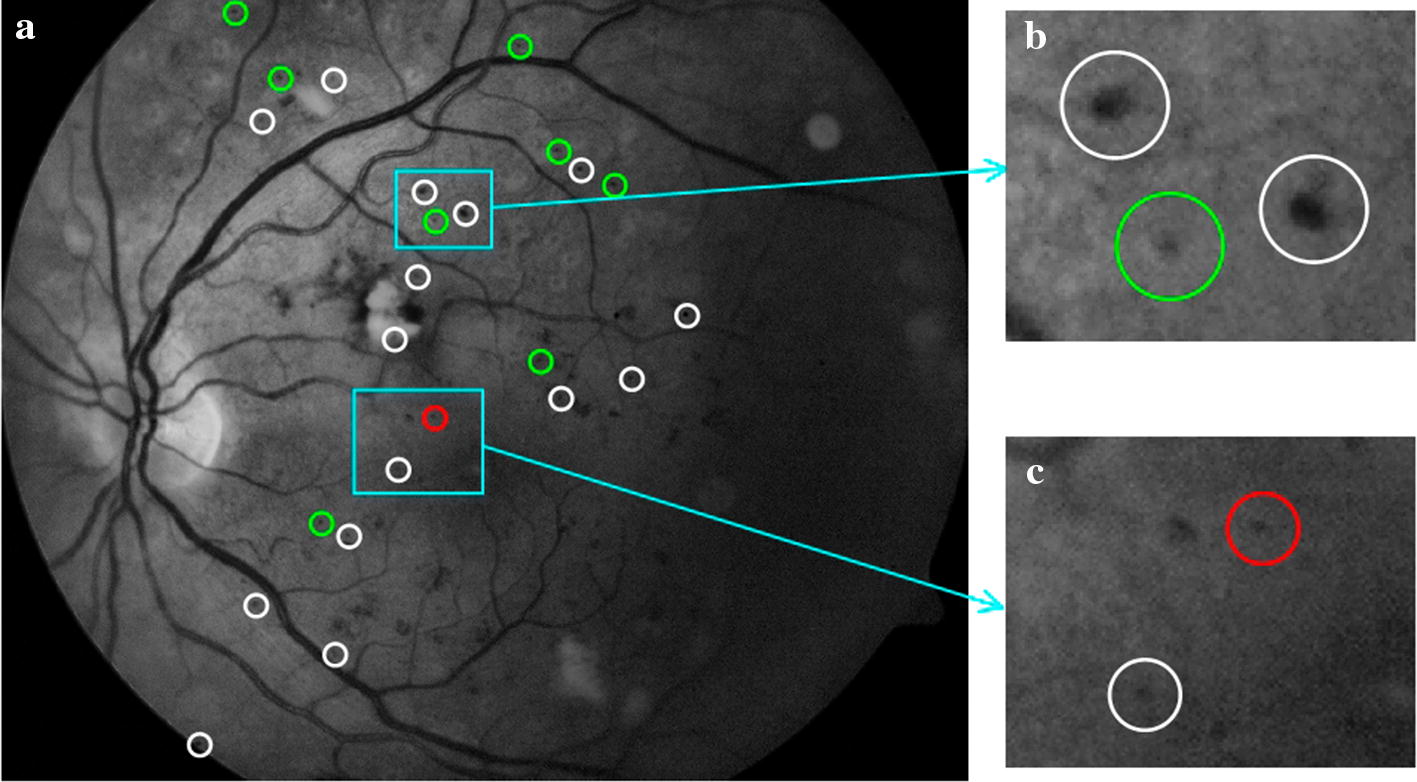
Lesion level evaluation for MA detection results on DIARETDB1 database. **a** Results of MA detection, where green circles indicate TPs, white circles indicate FPs, and the red circle indicates FN; **b** examples of TP and FP; **c** examples of FP and FN

Typical examples of MA detection results of our experiments on the two databases are shown in Figs. 6 and 7.

**Fig. 8:**
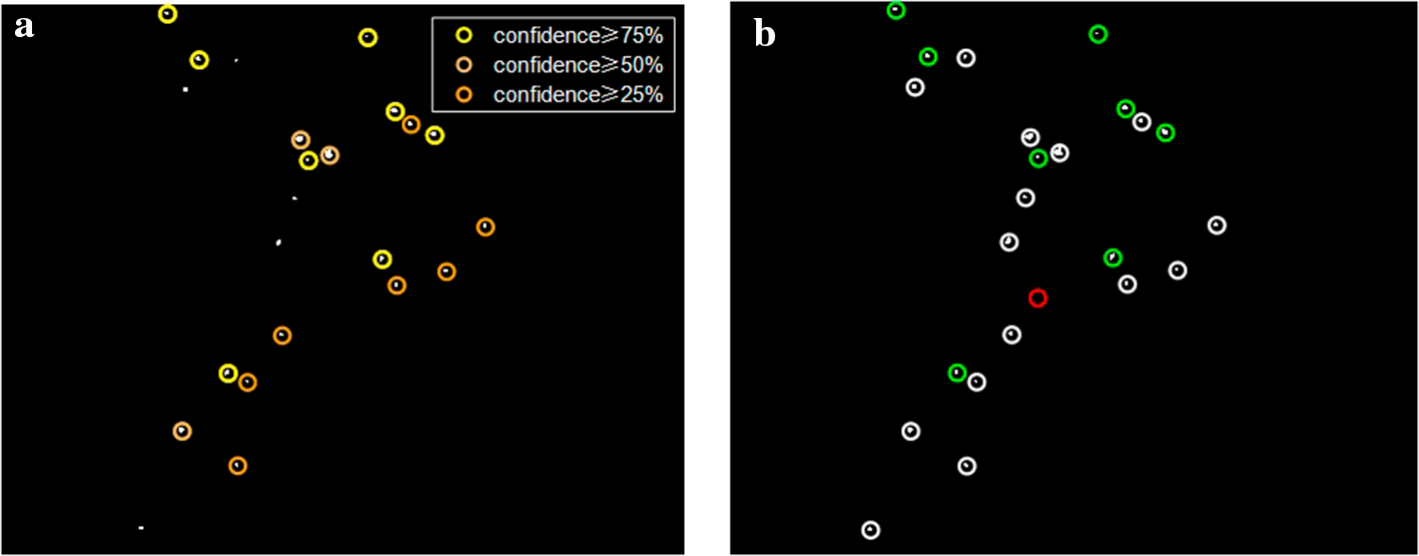
Analysis of MA detection results compared with ground truth on DIARETDB1 database. **a** MA detection results corresponding to different labeling confidences, where yellow circles indicate labels with confidence $\ge 75\%$, orange circles indicate labels with confidence $\ge 50\%$, and brown circles indicate labels with confidence $\ge 25\%$. **b** Evaluation of MA detection results with ground truth of confidence $\ge 75\%$, where green circles indicate TPs, white circles indicate FPs, and the red circle indicates FN, corresponding to Fig. 7

In DIARETDB1 database, MA detection results corresponding to different confidences are shown in different colored circles in Fig. 8a. The evaluation shown in Fig. 8b is based on the confidence $\ge 75\%$. As can be seen from Fig. 8, with a standard of ground truth confidence $\ge 75\%$, a part of FPs belong to those labeled with a confidence of less than $75\%$. For example, the two FPs shown in Fig. 7b have a confidence of $50\%$ (shown in Fig. 8a).

## Discussion

In this study, a method using machine learning based on directional local contrast is proposed for microaneurysms’ detection in color fundus images. The effectiveness of the method is proved through experiments on different databases.

### Advantages

The accurate segmentation of BV main structure could improve the accuracy of MA detection. It has been proven that using Jerman’s enhanced function can achieve the accuracy of $95.4\%$ in BV segmentation [33], with better performance compared to the widely used Hessian-based enhanced function proposed by Frangi [34]. For the features’ selection of MA candidate regions, directional local contrast (DLC) feature was creatively used in this study. The DLC of center point of each candidate patch was analyzed, which was the first time applied in MA detection and showed promising ability in differentiating MA (comparing Figs. 3 with Fig. 4). Through the analysis of characteristics of MA, it is found that the DLC of MA and non-MA patch is significantly different (Fig. 13). The accuracy of MA detection could be improved using the DLC feature. In addition, compared with existing methods, the proposed method performs better with relatively high accuracy and less computation time. As compared with methods using deep learning with CNN in high accuracy, whose main limitations are widely known that long training time and high requirement of computational resources (best with GPU), the proposed method is simpler and time saving.

### Typical misclassification analysis

Despite the good performance in MA detection achieved by the proposed algorithm, some misclassified examples affecting accuracy could be seen in Figs. 6 and 7. There are several causes for misclassification. For FPs, from the white circles in Figs. 6c and 7c, it can be seen that image noises could lead to erroneous MA detection. Throughout the experiment, most FPs were caused by image noises’ interference.

**Fig. 9:**
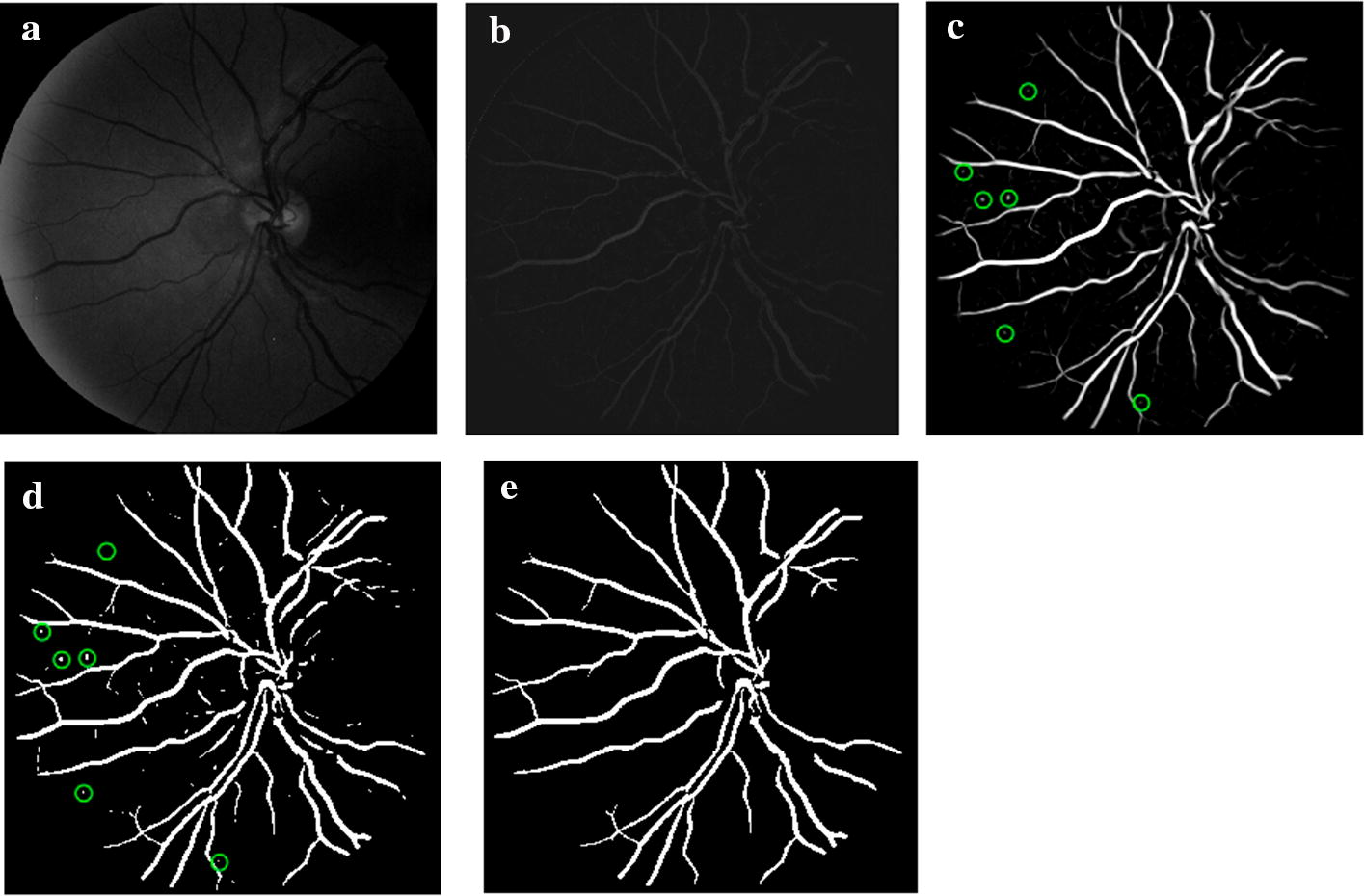
Process of blood vessels segmentation. **a** Green channel image; **b** result of shade correction ($I_\text {sc}$); **c** BV enhanced image (green circles indicate MAs); **d** preliminary BV segmentation (green circles indicate MAs); **e** final result of BV segmentation

For FNs, first, as shown by the red circle in Fig. 6b, the MA has an irregular shape and a relatively large area. It is a typical example that leads to misjudgment of the algorithm based on the shape features. As the shape features used to distinguish between MA and HM (shown in Table 6) that missed MA might be considered as HM by the classifier. Second, as shown by the red circle in Fig. 7c, this MA was missed in the process of MA candidate regions’ extraction. Although the applied method for extracting MA candidate regions was efficient, it might miss a small part of MA in candidate regions, which caused the increase of FNs in the final MA detection results and affected the overall accuracy of MA detection. The main reasons are as follows: in the first case, MA was attached to BV and removed with it. This case is relatively rare, because MA usually appears at the end of BV which was isolated from the main structure of BV and preserved as an independent slender structure during BV removing, as shown in Fig. 9d, e. In the second case, MA was discarded together with the attached slender segment of BV end in the step of removing slender structures. Thus, the threshold for removing slender structures is important. If it was set too small, some MAs would be removed together. If it was too large, some slender and small BV might still be left. In another case, when MA characteristics are not obvious, as shown by the red circle in Fig. 7c, this missed MA is too small and has low contrast to background, which looks more like image noise. It is difficult to judge whether it is MA.

**Table 6:** Descriptions of features for MA detection

Feature types	Symbols	Descriptions
Color	f1~2	Mean and standard deviation value of candidate patch in RGB color
	f3~4	Mean and standard deviation value of candidate patch in HSV color
	f5~6	Mean and standard deviation value of candidate patch in CIElab color
Grayscale	f7~8	Mean and standard deviation value of candidate patch in $I_\text {g}$
	f9~10	Mean and standard deviation value of candidate patch in $I_\text {CLAHE}$
DLC	f11~22	Directional local contrast (DLC) of the center point of each candidate region in $I_\text {CLAHE}$
Shape	f23~28	Area, Perimeter, Circularity, Eccentricity, Aspect ratio, and Solidity of each candidate region
Texture	f29~32	Entropy, Energy, Homogeneity and Skewness of candidate patch in $I_\text {CLAHE}$
Gaussian filter-based	f33~40	Mean and standard deviation value of candidate patch in corresponding Gaussian filtered result of $I_\text {CLAHE}$ when $\sigma$ is $\left[ 1,2,4,8\right]$
Gradient	f41~42	Mean gradient of candidate patch in $I_\text {CLAHE}$ (mean value of d*x* and d*y*)
	f43~44	Mean gradient on the boundary of each candidate region in $I_\text {CLAHE}$ (mean value of d*x* and d*y* on the boundary)

MA detection is a difficult task that there is disagreement between different experts in the diagnosis of MA, as shown in Fig. 8. As the DIARETDB1 database shows, from the observation of the image in Fig. 7, it can be seen that the two FPs in Fig. 7b are very similar to actual MA, while only two experts label them as MA (confidence of $50\%$). Because the MA structure is too small, unaided eye observation is also susceptible to image quality. From the viewpoint of clinical application, the algorithmic prediction and clinical diagnosis could be mutually complementary. If there is MA detected in an image, the ophthalmologist can further diagnose whether the image corresponds to DR by MA detection result. Therefore, some FN on lesion level will not affect the MA detection performance on image level, and has little impact on clinical diagnosis of ophthalmologist. Or a lower confidence level can be taken as ground truth for ophthalmologists to screen. Thus, the application of computer-aided MA detection in assisting clinical DR screening deserves further investigation.

### Limitations

1. One of the main limitations of this study is the interference of image noises on MA detection. It is difficult to distinguish MA and image noises in current method.
2. The scale of data for experiment is another important limitation. Two databases with totally 163 (74 in e-ophtha MA and 89 in DIARETDB1) images were used for test. The physiological and pathological factors that might affect MA detection could not be considered in this pilot study.

### Future works

For the current limitations above, in the subsequent research, the image denoising operation in preprocessing progress should be improved. In addition, to improve the generalization of the method, more databases that cover more heterogeneous samples are needed for as fully validation as possible, including real samples from hospitals for clinical validation.

## Conclusions

Making use of directional local contrast feature can improve the performance of machine learning method for MA detection. Naive Bayesian classification is more accurate than SVM and KNN in this study. The proposed MA detection algorithm showed good performance on the two databases with AUC of 0.87 and 0.86, FROC score of 0.374 and 0.210, respectively. In addition, compared with deep learning-based methods, the proposed method is simple and takes less computation time, of 29 s for image with $2544\times 1969$ resolution and 3 s for image with $1400\times 960$ resolution and 2.6 s with $1500\times 1152$ resolution. It is known that MA is important in DR progression, which is associated with DR severity and macular edema. Therefore, the proposed method with accurate detection of MA and less computation time is promising for clinical application of DR diagnosis and identification of eyes at risk of developing macular edema.

## Methods

This study uses machine learning based on directional local contrast to detect MA on preprocessed and segmented image patches.

First, preprocessing was used to enhance the image quality. Then blood vessels (BVs) were segmented using an improved enhanced function based on Jerman’s work [33]. With BVs eliminated, candidate regions of MA were then extracted. Next, processed images were divided into patches according to the location of the MA candidate regions. Finally, based on the features extracted, candidate patches were classified to differentiate the MA and non-MA. The process is illustrated in Fig. 2. To comprehensively evaluate the algorithm on different patient cohorts, the proposed method was validated on two independent databases (e-ophtha MA and DIARETDB1) on lesion level.

### Preprocessing

#### Shade correction

BVs, HMs and MAs all appear dark red in color fundus images (as shown in Fig. 1). To make the dark red regions more obvious, preprocessing progress was performed in the Field of View (FOV).

Compared to the other two channels, the green channel has higher contrast between target area and background, and therefore contains the most abundant information. Thus, the first step of preprocessing was to extract the green channel image $I_\text {g}$. Then, median filter on the green channel image $I_\text {g}$ was applied to obtain the background $I_\text {bg}$. The filter size was larger than the maximal BV width in the fundus image. In this study, the filter size of $50\times 50$ was selected. Finally, the result derived by the median filter $I_\text {bg}$ was subtracted from the green channel image $I_\text {g}$ to eliminate the background and achieve the image with the effect of shade correction, named $I_\text {eq}$. The main structure of BVs in $I_\text {eq}$ was clear. Other dark regions that include HM and MA in the original image were also highlighted in $I_\text {eq}$. But the overall image was dark with low contrast. To make the image clearer, the mean gray value of the green channel image $I_\text {g}$ in FOV was added on image $I_\text {eq}$ to maintain in the same gray range as the green channel image. As follows:

1 $$\begin{aligned} I_\text {sc}=I_\text {eq}+\text {mean}(I_\text {g}) \end{aligned}$$

The result of shade correction ($I_\text {sc}$) is shown as Fig. 9b where BV can be obviously observed.

#### Segmentation of blood vessels

For accurate MA detection, it is important to separate BV and MA which are similar in color and brightness. Thus, BVs were segmented and eliminated to minimize the influence on MA detection. The most widely used BV segmentation methods are machine learning [35] and deep learning [36, 37], which have high accuracy but are complicated and time-consuming. Inspired by Jerman et al.’s work [33], which was improved from the widely used method for BV enhancement of Frangi et al.’s work [34], this study constructed a response function to enhance BV by analyzing the eigenvalues of Hessian matrix, to achieve simple, fast, and accurate BV segmentation.

For a shade-corrected image $I_\text {sc}(x,y)$, first, Gaussian filter was applied to reduce the noise. Different $\sigma$ values were selected for Gaussian filter to obtain different enhancement functions, and the largest response function was selected to derive BV enhanced image. The value range of $\sigma$ was $3\sim 6$ and the step was 1 in this study.

2 $$\begin{aligned} G(x,y)=\frac{1}{2\pi \sigma ^2}e^{-\frac{x^2+y^2}{2\sigma ^2}} \end{aligned}$$

The second-order partial derivatives of *x* and *y* on the filtered image were computed respectively, then convolved with $I_\text {sc}(x,y)$, getting $L_\text {xx}$, $L_\text {xy}$, $L_\text {yy},$ respectively. The Hessian matrix *H* was composed as follows:

3 $$\begin{aligned} H=\left[ \begin{array}{rcl} L_\text {xx} &{} L_\text {xy} \\ L_\text {xy} &{} L_\text {yy}\ \end{array} \right] . \end{aligned}$$

Then, two eigenvalues $\lambda _1$ and $\lambda _2$ could be obtained from Hessian matrix *H* as follows, where tmp represented an intermediate variable.

4 $$\begin{aligned} \text{tmp}=\sqrt{(L_\text {xx}-L_\text {yy})^2+4*L_\text {xy}^2} \end{aligned}$$

5 $$\begin{aligned} \mu _1=0.5*(L_\text {xx}+L_\text {yy}+\text{tmp}) \end{aligned}$$

6 $$\begin{aligned} \mu _2=0.5*(L_\text {xx}+L_\text {yy}-\text{tmp}) \end{aligned}$$

If $|\mu _1|\le |\mu _2|$, then $\lambda _1=\mu _1,\lambda _2=\mu _2$. Otherwise, $\lambda _1=\mu _2,\lambda _2=\mu _1$ (that is, to ensure $|\lambda _2|\ge |\lambda _1|$). $\lambda _2$ was normalized as follows:

7 $$\begin{aligned} \lambda _{\rho }=\left\{ \begin{array}{lll} \text {max}(\lambda _2) &{} &{}{\lambda _2>0}\\ 0 &{} &{}\text {else}\end{array} \right. . \end{aligned}$$

Enhancement function was calculated as follows:

8 $$\begin{aligned} \text {Enhancement}=\left\{ \begin{array}{lll} 0 &{} &{}{{\lambda _2}\le {0}\cup {\lambda _{\rho }}\le {0}}\\ 1 &{} &{}{{\lambda _2}\ge {\frac{\lambda _{\rho }}{2}}>0}\\ {\lambda _2}^2(\lambda _{\rho }-\lambda _2)(\frac{3}{\lambda _2+\lambda _{\rho }})^3 &{} &{}\text {else}\\ \end{array} \right. . \end{aligned}$$

The BV enhancement result is shown in Fig. 9c. Because the BV enhancement process might also enhance some other dark red regions in the original image, MA might be enhanced. The green circles in Fig. 9c indicate the MAs contained in the BV enhancement image.

First, the Ostu’s adaptive threshold method was used to binarize the BV enhanced image in order to obtain the preliminary BV segmentation result, as shown in Fig. 9d. MAs might be contained in the result, shown as the green circles in Fig. 9d. To remove MAs from BV segmentation result, small regions were excluded to obtain the final BV main structure segmentation result, as shown in Fig. 9e. Compared with Fig. 9d, MAs have been removed.

#### Contrast enhancement and noise reduction

Due to the tiny structure of MA, the accuracy of MA detection is sensitive to image quality. Therefore, further preprocessing was performed to improve the image quality.

**Fig. 10:**
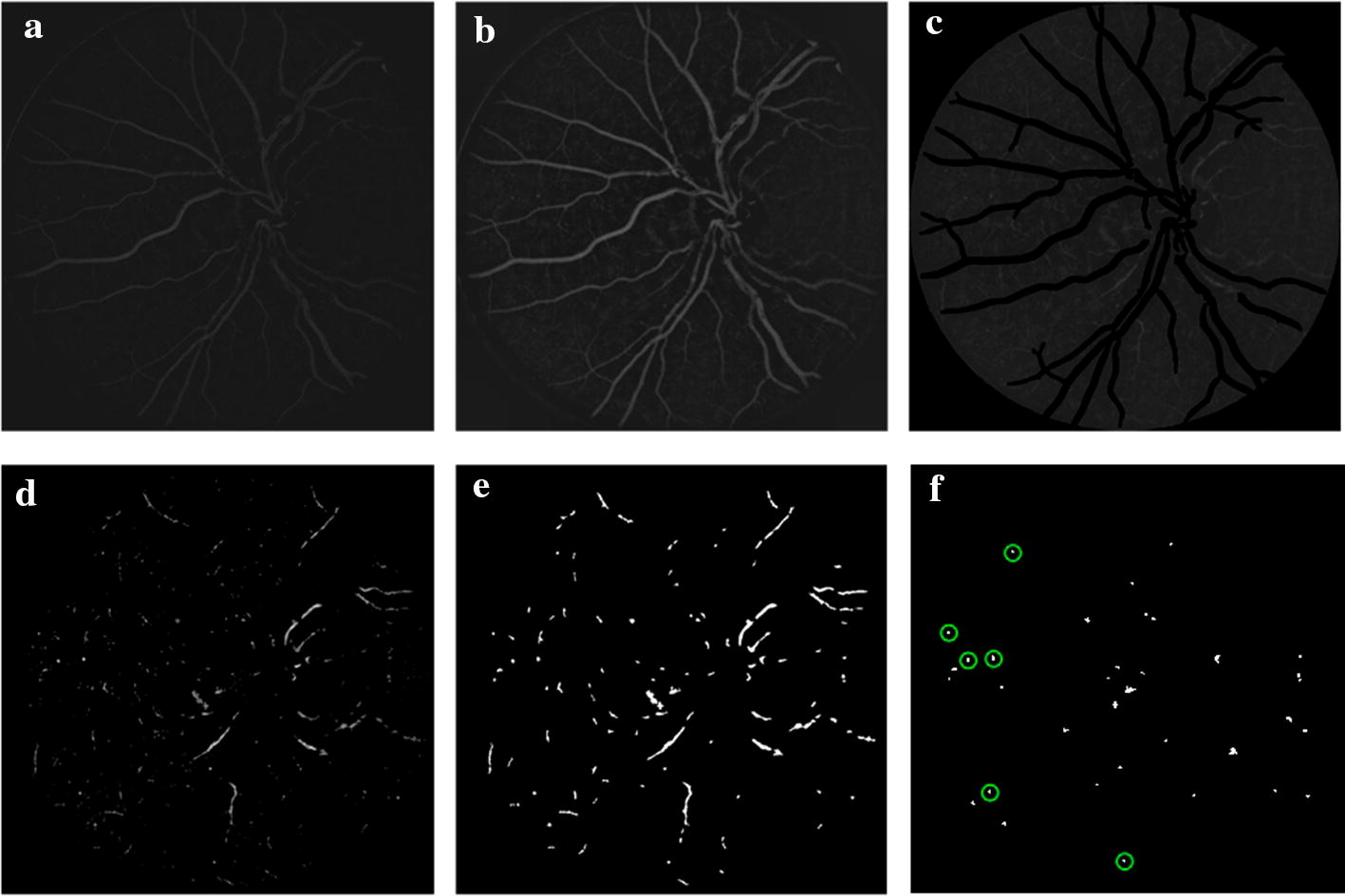
Process of MA candidate regions extraction. **a** Result of shade correction ($I_\text {sc}$); **b** result of preprocessing ($I_\text {gauss}$); **c** result of BV removal ($I_\text {gsBV0}$); **d** result of contrast stretch ($I_\text {CS}$); **e** preliminary MA candidate regions ($I_\text {can1}$); **f** final result of MA candidate regions ($I_\text {can}$, green circles indicate ground truth of MAs)

To enhance the contrast between background and highlighted dark regions (including BVs, HMs, and MAs) in the shade-corrected image $I_\text {sc}$, the image was processed with contrast-limited adaptive histogram equalization (CLAHE) method. A $7\times 7$ Gaussian filter was then applied to reduce the influence of noise. The result ($I_\text {gauss}$) is shown in Fig. 10b.

#### Blood vessels removal

To eliminate the effects of BV on MA detection, on the filtered image ($I_\text {gauss}$), the gray value of the corresponding position of the segmented BV was directly set to zero, obtaining $I_\text {gsBV0}$ as shown in Fig. 10c.

### Extraction of MA candidate regions

In the previous step of BV removal, the gray value of BV has been directly set to zero. To further make all the other parts black except for the MA candidates, first, the mean gray value of original green channel image in FOV (see Eq. 1) was subtracted from $I_\text {gsBV0}$. Then contrast stretch was used, obtaining $I_\text {CS}$ as shown in Fig. 10d. Finally, the Ostu’s adaptive threshold method was used to binarize to obtain the preliminary MA candidate regions $I_\text {can1}$, as shown in Fig. 10e.

In BV segmentation, the small regions were excluded to ensure that candidate regions of MA were not included in the segmented BV. As a result, the preliminary MA candidate regions contained some small BV segments which appear as slender structures in Fig. 10e.

Then, the connected component analysis and shape characteristics were used to refine the results of MA candidates. Since MAs appear as dots, the remaining slender structures could be excluded by the ratio (named *R*) of the length of the major axis and the minor axis of each candidate region (or connected component). If the ratio *R* of a candidate region exceeds the set threshold, the region would be discarded.

The threshold was set based on the mean value of the ratio *R* of all connected components in the preliminary MA candidate regions. Considering the proportion of the number of the remaining slender structure, through several experiments, 1.2 was finally selected to multiply the mean value, formulated as follows:

9 $$\begin{aligned} \text {Threshold}=1.2*\frac{1}{n}\sum _{i=1}^{n}{R(i)}, \quad i=1,2,\ldots ,n, \end{aligned}$$

where *n* is the total number of connected components in the preliminary MA candidate regions.

Finally, in remaining candidate regions, those with area larger than 500 pixels were discarded, since MAs are often much smaller (maximum MA with $100\sim 300$ pixels in existing works [22, 23, 28, 38], and 361 pixels labeled in e-ophtha MA database). The final result of MA candidate regions $I_\text {can}$ is shown in Fig. 10f, where green circles indicate ground truth of MAs.

### Image patches

MA is difficult to observe and detect at the whole image level due to its tiny structure. Therefore, the detection of MA was based on image patches.

**Fig. 11:**
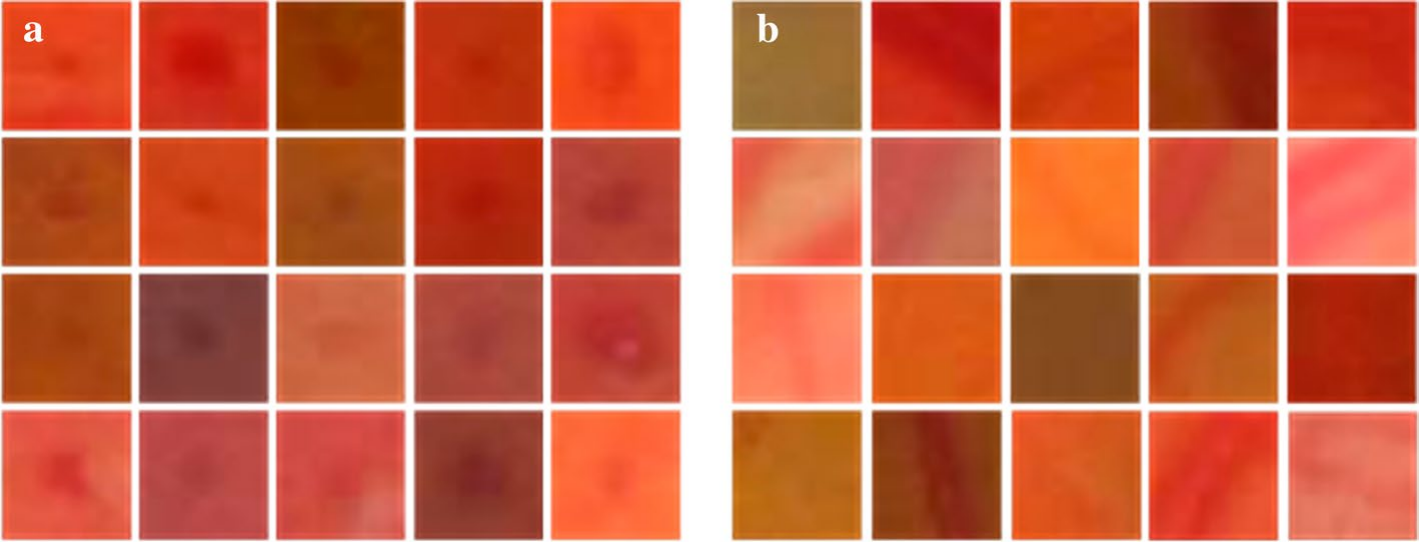
Examples of patches. **a** MA patches; **b** non-MA patches

All images in training set and test set were divided into $25\times 25$ patches according to the location of candidate regions, and a suitable classification method was selected to determine whether each test patch contains MA, so as to realize MA detection. MA patches and non-MA patches are shown in Fig. 11.

### Features extraction and classification

**Fig. 12:**
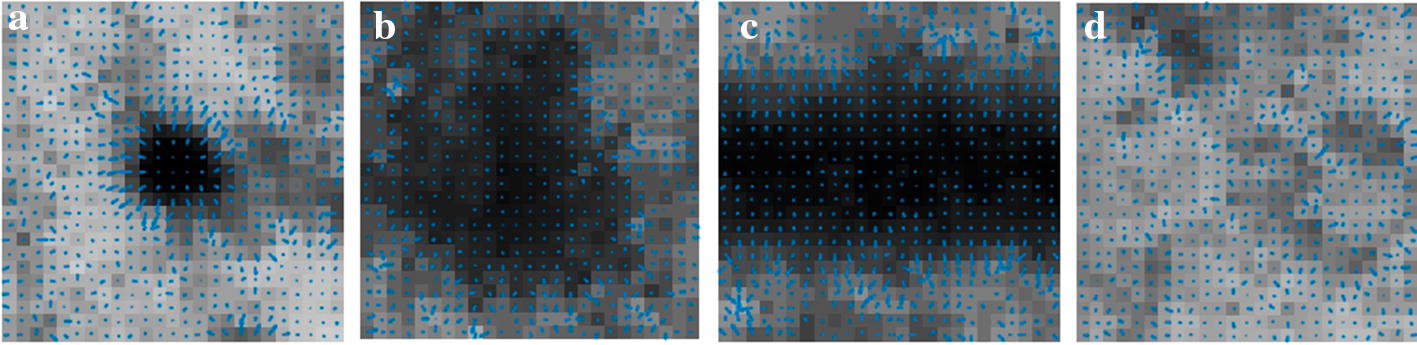
Comparison of different structures in 25×25 patch. **a** MA; **b** HM; **c** BV; **d** background

Although the interference of BV on MA detection was eliminated, in addition to image noises in the obtained candidate regions, there might also be HMs. To further separate the true MA from the candidate regions, it is necessary to analyze the difference between MA and HM. As shown in Figs. 1 and 12, MAs appear as isolated dark red dots with clear borders, while HMs appear as dark red regions of different sizes and shapes with blurred borders. Thus, using shape features or combined with gradient features could distinguish MA and HM.

Moreover, for each MA candidate patch, the directional local contrast (DLC) of its center point *p* was calculated to distinguish MA and other structures. Assuming that the gray level of the center point *p* is $I_p$, the *DLC* of the point *p* along the direction angle $\theta$ is defined as

10 $$\begin{aligned} DLC_{p(\theta )}= & {} \frac{I_p-{\overline{I}}_{p(\theta )}}{{\overline{I}}_{p(\theta )}}, \end{aligned}$$

11 $$\begin{aligned} {\overline{I}}_{p(\theta )}= & {} \frac{1}{r}\sum _{q\in {N_{p(\theta ,r)}}}{I_q}, \end{aligned}$$

where ${\overline{I}}_{p(\theta )}$ is the mean gray value of neighboring pixels of point *p* along the direction angle $\theta$, *r* the radius of neighboring region, and $N_{p(\theta ,r)}$ the set of neighboring pixels of point *p* along the direction angle $\theta$ within the radius *r*:

12 $$\begin{aligned} N_{p(\theta ,r)}=\left\{ (x_q,y_q)|x_q=x_p+k\cos \theta ,y_q=y_p+k\sin \theta , \quad k=1,2,\ldots ,r\right\} . \end{aligned}$$

**Fig. 13:**
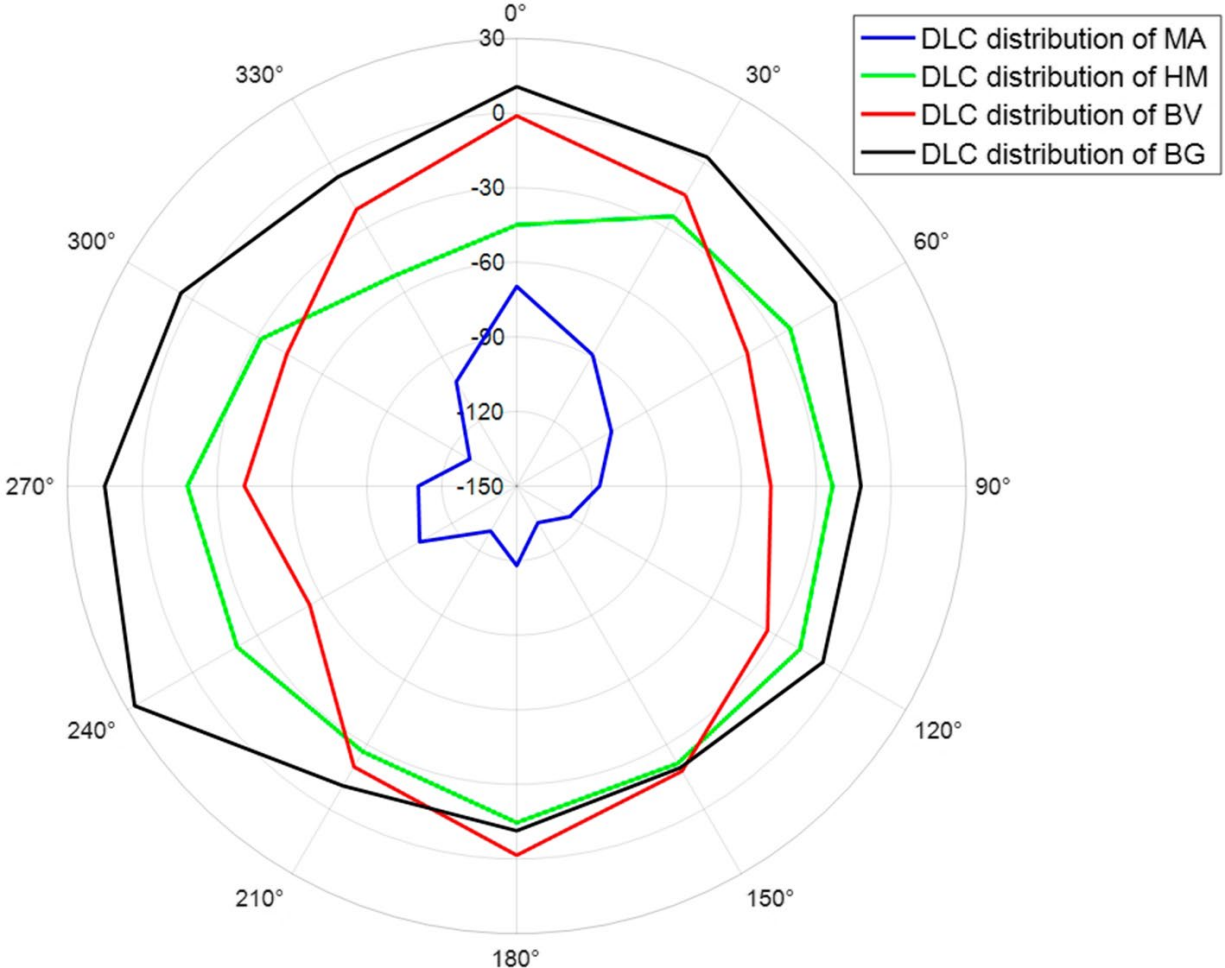
DLC distribution on different structures shown in Fig. 12, where radius indicates the DLC value along the direction angle $\theta$

Finally, the obtained *DLC* vector of point *p* is

13 $$\begin{aligned} DLC_p=(DLC_{p(\theta _1)},DLC_{p(\theta _2)},\ldots ,DLC_{p(\theta _n)}), \end{aligned}$$

where *n* is the number of angles, 12 was selected, that is, $360^{\circ }$ was divided into 12 angles with a step of $30^{\circ }$. And the selected value of *r* was 12, which was half the patch size. DLC indicates the local contrast characteristics in a neighborhood. A negative DLC value indicates that the gray value of the current pixel is lower than that of the neighborhood. Therefore, DLC could be used to differentiate the structures with different local contrast. Figure 13 shows DLC distribution of center point of each region of MA, HM, BV and background (BG) shown in Fig. 12.

As shown in Fig. 13, the difference between MA and other regions (HM, BV, BG) in DLC distribution is obvious. The analysis of variance (ANOVA) results showed that the DLC of MA is significantly different from those of HM, BV, and BG (p$<0.05$ for all).

With the aim of distinguishing between true MA and non-MA, totally seven types of features including color, grayscale, DLC, shape, texture, Gaussian filter-based, and gradient were selected for each candidate (or patch). The details are shown in Table 6, where $I_\text {CLAHE}$ indicates the result of CLAHE on the green channel.

**Area** is the total number of pixels in candidate region, that is the number of white pixels of each connected component in Fig. 10f.

**Perimeter** is the total number of boundary pixels that surround candidate region.

**Circularity** of each candidate region is defined as $\text{Circularity}=\frac{\text{Perimeter}^2}{4*\pi *\text{Area}}$

**Aspect ratio** is the ratio of the length of major axis to minor axis of candidate region.

**Eccentricity** is the ratio of the distance between the two focal points of the circumscribed ellipse of candidate region (focal length 2*c*) to its major axis length (2a), and it is equal to 0 for a circular region, as $\text{Eccentricity}=\frac{c}{a}$

**Solidity** is the ratio of area of candidate region to its convex hull area (ConA), as $\text{Solidity} = \frac{{\text{Area}}}{{\text{ConA}}}$

**Entropy** indicates image randomness or texture complexity, representing the clustering characteristics of gray distribution. With *n* different gray values in the image and proportion *p*(*i*) of each gray value *i*, it is defined as $\text{Entropy}=-\sum _{i=1}^{n}({p(i)*\text {log}_2{p(i)}}),i=1,\ldots ,n$. When gray level of image is uniform, entropy can reach the maximum.

**Energy** reflects the uniformity of image gray distribution and texture thickness, and it is the sum of square of element values of gray-level co-occurrence matrix (GLCM). With *P* indicating GLCM, it is defined as $\text{Energy}=\sum _{i,j}{P(i,j)^2}$

**Homogeneity** reflects the tightness of distribution of elements to the diagonal in GLCM, defined as $\text{Homogeneity}=\sum _{i,j}\frac{P(i,j)}{1+|i-j|}$

**Skewness** reflects the asymmetry of gray value distribution of image. For input gray values *x* of pixels, with $\mu$ and $\sigma$ representing the mean and standard deviation of the input *x*, respectively, and *E* representing expectation, it is defined as $\text{Skewness}=\frac{E(x-\mu )^3}{\sigma ^3}$

As for gradient features, d*x* and d*y* indicate the gradient in the horizontal and vertical direction, respectively. Gradient at MA boundary has an abrupt change. As shown in Fig. 12a, gradient vector on the boundary of MA is divergent, where the absolute value of gradient is large. Whereas the absolute value of gradient at the center of MA is small. As shown in Fig. 12b, the distribution of gradient vector in the patch of HM is relatively messy. It can be seen from Fig. 12 that the difference in gradient vector distribution is obvious between MA and other structures. Therefore, features based on gradient can be used as reference to distinguish MA from image patches of candidate regions.

### Machine learning algorithms for classification

After all the 44 features extracted from each MA candidate patch, machine learning technique would be used to classify each patch to MA or non-MA.

As aforementioned, a variety of classification methods were used in automatic DR detection, including MA detection with Naive Bayesian [16], random forest [17], support vector machine [18, 26, 39] and K-nearest neighbor [23, 24]. Three classifiers were selected for MA detection and compared the results in this work: NB, KNN and SVM.

#### Naive Bayesian

NB classifier is supervised learning technique based on Bayesian theory. It assumes that features are independent (naive) of each other. NB calculates the prior probability of each class and the conditional probability of each class for each feature from the training samples. For a test sample to be classified, Bayesian theory is used to calculate the posterior probability of test sample belonging to each class, and the class with the maximum posterior probability will be chosen. NB is simple and powerful therefore has been widely used in machine learning algorithms of spam processing, document classification, disease diagnosis and other aspects.

#### K-nearest neighbor

KNN is a very special kind of machine learning algorithm because it does not have a learning process in a general sense, which named lazy learning. Lazy learning is also called mechanical learning due to its high dependence on training samples. Mechanical learning does not build a model, so KNN is a non-parametric supervised learning method. For a test sample to be classified, KNN calculates the distance between the test sample and all training samples, selects the K samples closest to the test sample from the training dataset, and according to the majority-voting rule, the test sample is classified into the class that the more samples of K nearest neighbors belong to.

#### Support vector machine

SVM is a classic supervised learning algorithm in the field of machine learning, mainly used to solve the problem of data classification. SVM algorithm aims to find a maximum-margin hyperplane to correctly divide the training samples to different categories. For linearly inseparable problems, kernel functions are used to map the input features into the higher-dimensional feature space where a linear separation is possible. Kernel functions used in SVM classifier mainly include linear, sigmoid, polynomial and radial basis function (RBF).

### Experiment materials

The fundus images of two available public retinal image databases (e-ophtha MA [40] and DIARETDB1 [41]) were used for experiments.

In e-ophtha MA database, there are four image resolutions ranging from $1440\times 960$ to $2544\times 1696$ pixels with $45^{\circ }$ FOV. The e-ophtha MA database contains 233 images without MAs, and 148 images MAs, which are manually annotated by an ophthalmologist and confirmed by another. The 148 images with totally 1306 MAs were used in this study for experiments.

DIARETDB1 database contains 89 color fundus images with resolution of $1500\times 1152$ pixels and $50^{\circ }$ FOV. Four medical experts marked MA in DIARETDB1 database independently. Considering the disagreement between the four experts annotations, we use the annotations with confidence $\ge {75\%}$ (at least three of the four experts agree that there is an MA) as standard for MA labeling, and there are 182 MAs with confidence $\ge {75\%}$.

74 images were selected from e-ophtha MA database and segmented to derive the training set. The segmented patches on MA locations and non-MA candidate patches were positive and negative samples, respectively, making up the training set.

The remaining 74 images in e-ophtha MA database and the whole DIARETDB1 database were used as test set to verify the performance of the proposed MA detection method.

### Experiment procedure

From each patch of the training set, the aforementioned 44 features were extracted to train the classifier. Then, the trained classifier was used on each test patch to determine whether the candidate was MA based on the 44 features extracted, so as to achieve the purpose of MA detection.

NB, KNN and SVM with RBF kernel function were used to perform the experiment and compared to choose the best performed classifier, and then the results of MA detection were evaluated on lesion level.

### Evaluation

For the problems of class-imbalance, the receiver operating characteristic (ROC) curve can better evaluate the performance of the classifier. In this study, the number of MAs in candidate regions is much smaller than that of Non-MAs. Therefore, **ROC** curve and its corresponding Area Under Curve (**AUC**) as well as free-response ROC (**FROC**) curve were used to evaluate the proposed MA detection method. The closer ROC curve is to the upper left corner, the more convex the ROC curve is, the larger the value of AUC is, the better the algorithm performs.

The abscissa of ROC curve is false-positive rate (FPR), which is the proportion of negative samples with positive classification results to all negative samples. The ordinate is Sensitivity, also named true-positive rate (TPR), which is the proportion of positive samples with positive classification results to all positive samples. The two variables corresponding were calculated as follows:

14 $$\begin{aligned} \text{FPR}\,=\, & {} 1-\text{Specificity}=1-\frac{\text{TN}}{\text{TN}+\text{FP}} \end{aligned}$$

15 $$\begin{aligned} \text{TPR}= \, & {} \text{Sensitivity}=\frac{\text{TP}}{\text{TP}+\text{FN}} \end{aligned}$$

TP indicates that MA is correctly predicted. TN indicates that non-MA is correctly predicted. FP indicates that non-MA is erroneously predicted as MA. And FN indicates that MA is erroneously predicted as non-MA.

Compared with ROC curve, FROC curve has the same Sensitivity on the ordinate and the false-positive per image (FPI) on the abscissa.

As both databases provide the annotation of MA on lesion level, evaluation of the results was performed on lesion level. As aforementioned, the ground truth of DIARETDB1 database with confidence $\ge {75\%}$ was used as the standard for evaluation of MA detection results.

First of all, ROC curve and AUC were used to evaluate the performance of three classifiers on MA detection, so as to select the best performed classifier, and further analyze its results of MA detection.

Next, FROC curve and FROC **score** were used to evaluate the performance of the proposed MA detection method and be compared with existing algorithms.

Then, average computation time per image of the whole experiment and of each processing step was recorded and compared with existing algorithms.

Last, typical examples of MA detection results evaluated on lesion level were analyzed.

## Data Availability

The data used or analyzed during the study are available from the corresponding author on reasonable request.

## Abbreviations

DR: Diabetic retinopathy
DLC: Directional local contrast
AUC: Area under curve
ROC: Receiver operating characteristic
FROC: Free-response ROC
NB: Naive Bayesian
SVM: Support vector machine
KNN: K-nearest neighbor
FFA: Fundus fluorescein angiography
OCT: Optical coherence tomography
MAs: Microaneurysms
HMs: Hemorrhages
HEs: Hard exudates
CWSs: Cotton wool spots
CAD: Computer-aided diagnose
RF: Random forest
MKL: Multiple-kernel learning
BV: Blood vessel
PCA: Principal component analysis
ADOBoosting: Adaptive over-sampling boosting
RUSBoosting: Random under-sampling boosting
LBP: Local binary pattern
DDL: Discriminative dictionary learning
MFFDL: Multi-feature fusion dictionary learning
CNN: Convolutional neural networks
FN: False-negative
FP: False-positive
TN: True-negative
TP: True-positive
FPR: False-positive rate
TPR: True-positive rate
FPI: False-positive per image
FCN: Fully convolutional neural networks
FOV: Field of view
CLAHE: Contrast-limited adaptive histogram equalization
ANOVA: Analysis of variance
BG: Background
GLCM: Gray-level co-occurrence matrix
RBF: Radial basis function.
